# Observation of Termination-Dependent Topological Connectivity in a Magnetic Weyl Kagome Lattice

**DOI:** 10.1021/acs.nanolett.3c02022

**Published:** 2023-08-28

**Authors:** Federico Mazzola, Stefan Enzner, Philipp Eck, Chiara Bigi, Matteo Jugovac, Iulia Cojocariu, Vitaliy Feyer, Zhixue Shu, Gian Marco Pierantozzi, Alessandro De Vita, Pietro Carrara, Jun Fujii, Phil D. C. King, Giovanni Vinai, Pasquale Orgiani, Cephise Cacho, Matthew D. Watson, Giorgio Rossi, Ivana Vobornik, Tai Kong, Domenico Di Sante, Giorgio Sangiovanni, Giancarlo Panaccione

**Affiliations:** † Department of Molecular Sciences and Nanosystems, Ca’ Foscari University of Venice, 30172 Venice, Italy; ‡ Istituto Officina dei Materiali, Consiglio Nazionale delle Ricerche, Trieste I-34149, Italy; ¶ Institut für Theoretische Physik und Astrophysik and Würzburg-Dresden Cluster of Excellence ct.qmat, Universität Würzburg, 97074 Würzburg, Germany; § School of Physics and Astronomy, University of St Andrews, St Andrews KY16 9SS, United Kingdom; ∥ Elettra Sincrotrone Trieste S.C.p.A. S. S. 14, km 163.5, 34149 Trieste, Italy; ⊥ Department of Physics, University of Arizona, Tucson, Arizona 85721, United States; # Dipartimento di Fisica Universitá di Milano, Via Celoria 16, Milano 20133, Italy; @ Diamond Light Source, Harwell Campus, Didcot OX11 0DE, United Kingdom; △ Department of Physics and Astronomy, University of Bologna, 40127 Bologna, Italy; ∇ Center for Computational Quantum Physics, Flatiron Institute, 162 5th Avenue, New York, New York 10010, United States; ¶¶ Università degli studi di Trieste Via A. Valerio 2, 34127 Trieste, Italy; §§ Forschungszentrum Juelich GmBH PGI-6Leo Brandt Strasse, 52425 Juelich, Germany

**Keywords:** magnetic kagome, spin−orbit coupling, topology, surface states

## Abstract

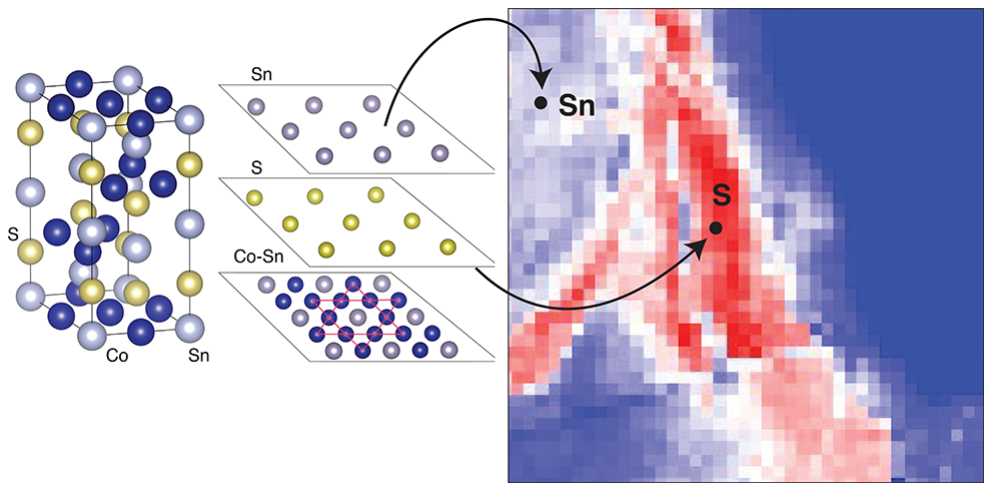

Engineering surfaces and interfaces of materials promises great potential in the field of heterostructures and quantum matter designers, with the opportunity to drive new many-body phases that are absent in the bulk compounds. Here, we focus on the magnetic Weyl kagome system Co_3_Sn_2_S_2_ and show how for the terminations of different samples the Weyl points connect differently, still preserving the bulk-boundary correspondence. Scanning tunneling microscopy has suggested such a scenario indirectly, and here, we probe the Fermiology of Co_3_Sn_2_S_2_ directly, by linking it to its real space surface distribution. By combining micro-ARPES and first-principles calculations, we measure the energy-momentum spectra and the Fermi surfaces of Co_3_Sn_2_S_2_ for different surface terminations and show the existence of topological features depending on the top-layer electronic environment. Our work helps to define a route for controlling bulk-derived topological properties by means of surface electrostatic potentials, offering a methodology for using Weyl kagome metals in responsive magnetic spintronics.

The discovery almost two decades ago of oxide heterostructures has provided evidence of novel electronic and structural phases that are absent in the bulk compounds but emerge at the interface between them.^1−3^ To date, heterojunctions based on transition-metal oxides and dichalcogenides as well as on van der Waals systems have been proposed and in some cases also fabricated in the context of spintronics, superconducting devices, solar cells, and low-volatility electronics.^4,5^ In topological quantum materials, the so-called topological protection is believed to be the key for taking interface electronics to a higher level of technological potential. Such developments depend critically on our ability to control the properties of topological states at surfaces or interfaces. The bulk-boundary correspondence, a consequence of the nontrivial topology of band structures, implies the existence of protected electronic states at surfaces, edges, and hinges. However, their precise arrangement, their electronic connectivity, and the spatial extension of their wave functions are less universal and crucially depend on various materials’ properties. For this reason, the knowledge of the surface states in classes of topological compounds is critical for future applications. Magnetic Weyl systems with layered kagome structure belong to this category, and their surface electronic properties have been intensely studied. Our study provides a solid and comprehensive view of the effect of the surface environment on the electronic properties of Co_3_Sn_2_S_2_.

The kagome lattice is a planar network of regular hexagons surrounded by corner-sharing triangles. Materials with a two-dimensional kagome atomic arrangement have been in the spotlight for the study of spin frustration, because of their peculiar geometry.^6−11^ More recently, theoretical predictions, followed by experimental verification, have established kagome lattices as a new breed of correlated topological phases, with the mutual existence of high-electron density flat bands, itinerant graphene-like Dirac states, and the appearance of charge density wave and superconducting phases.^12−18^ Additionally, magnetic kagome compounds show unusual transport phenomena, including the anomalous Hall effect and angle,^19−24^ which are ultimately attributed to the local momentum enhancement of the Berry curvature.^21,25−31^ In time-reversal broken systems, if the spin–orbit coupling (SOC) is sufficiently strong, orbital mixing can also promote the appearance on nontrivial Chern phases that are topologically invariant.^32,33^

Of particular interest are magnetic Weyl systems with a kagome structure. In these materials, the combination of the crystal field, breaking of time-reversal symmetry (TRS), and SOC is responsible for lifting most of the electronic energy degeneracies in the Brillouin zone (BZ). This is not the case for the Weyl points (WPs) (Figure 1A), topologically protected crossings of Bloch eigenvalues in momentum space (*k*-space), which are monopoles of Berry curvature.^34−37^ The latter are responsible for the anomalous Hall effect in several quantum materials.^38−42^ In-gap topological surface states connect the WPs and give rise to arc-like features, the so-called Fermi arcs (Figure 1A), which participate in the topological properties and transport of Weyl quantum systems. The distribution of the Berry phase in reciprocal space is therefore crucial, and the bulk-surface connectivity is tightly bound to it. In this work, we consider Co_3_Sn_2_S_2_ (Figure 1B), which is designed to support SOC and TRS breaking-derived Fermi arcs and has been suggested to host a termination-dependent topological bulk-surface connectivity.^27,43−46^

**Figure 1:**
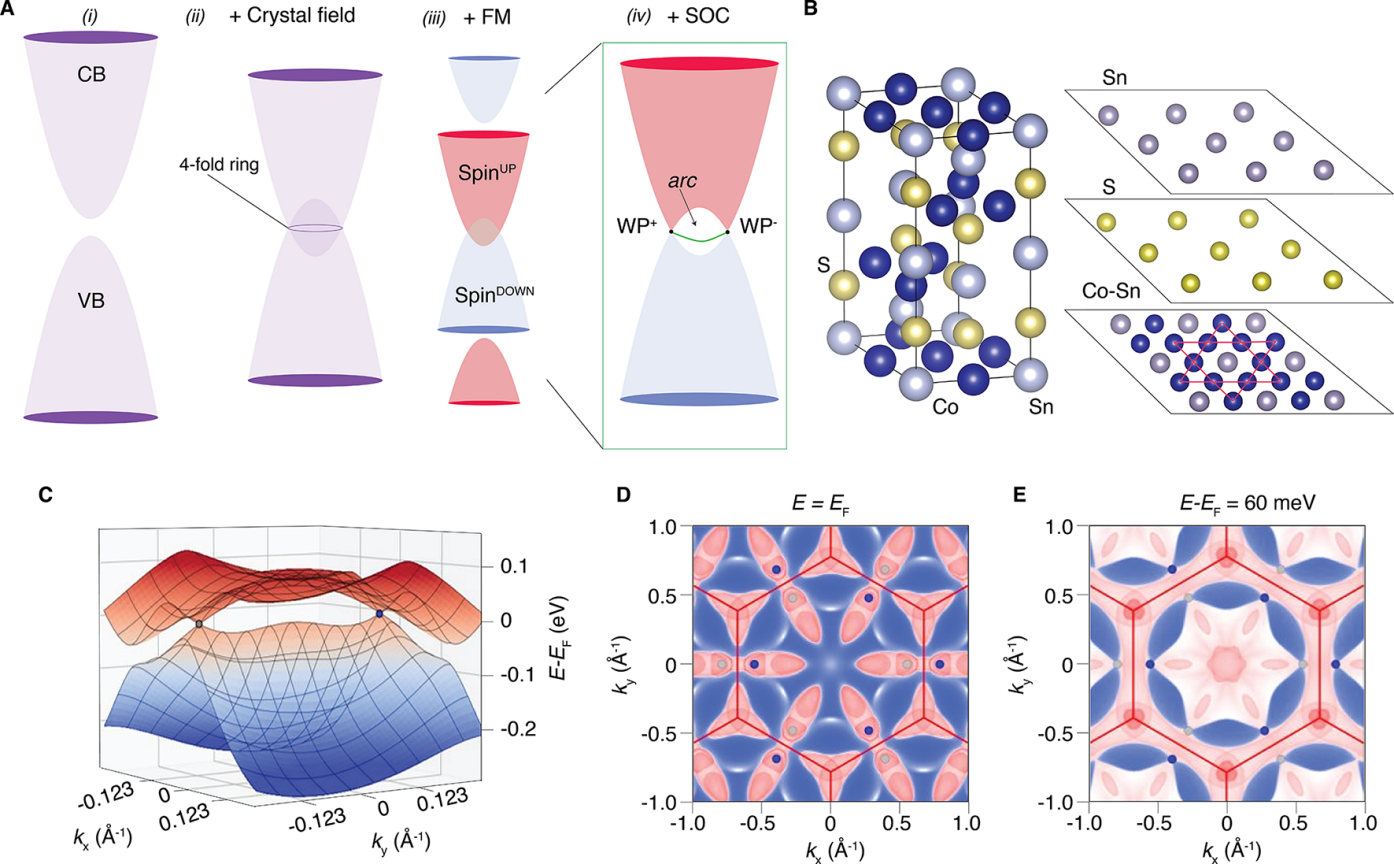
(A) Fermi arc formation mechanism in Co_3_Sn_2_S_2_. (i) The conduction and valence bands (CB and VB, respectively) intersect (ii) as a consequence of the crystal field and form a 4-fold degenerate nodal ring. (iii) The ferromagnetism (FM) splits the bands with opposite spins, and (iv) the SOC gaps the electronic structure but two points, the WPs. The WPs are connected by surface states that form arcs in the Fermi surface. (B) Crystal structure of Co_3_Sn_2_S_2_ shown for a single unit cell. The Co–Sn plane has the Co atoms arranged in a kagome lattice; S and Sn form planes in a triangular fashion. (C) Calculated electronic structure around two adjacent Weyl points. (D) Bulk Fermi surface map obtained with the semi-infinite Green’s function method corresponding to projections into the *k*_*z*_ = 0 plane. (E) Bulk constant energy surface map (60 meV above the Fermi level) of Co_3_Sn_2_S_2_ highlighting the position of the Weyl points. Increasing spectral intensity is indicated by the color changing from blue to white to red.

Co_3_Sn_2_S_2_ has three possible cleavage planes, Sn, S, and Co–Sn, as shown in Figure 1B. Previously, scanning tunneling microscopy (STM) showed clear evidence for the presence of such multiple surface terminations obtained after the cleavage of the samples.^43,44^ In addition, the interference pattern of the quasiparticle density of states revealed by the STM tip (so-called quasiparticle interference) demonstrates different scattering channels in momentum space for different terminations.^43^ This result, supported by density functional theory (DFT) calculations, hints at the possibility that connectivity of the Fermi arcs in Co_3_Sn_2_S_2_ is affected by the local surface environment. Previous photoelectron spectroscopy studies investigated the evolution of this system across the transition temperature^47^ and have suggested the existence of a termination-dependent topological connectivity. However, the absence of marked spectroscopic features attributable to the surface in the core levels makes it difficult to clearly understand the role of the surface in topological connectivity. Here, we investigate this fundamental issue by studying both the spectra and the Fermi surfaces of Co_3_Sn_2_S_2_ for Sn and S termination and elucidate how the WPs are connected by means of DFT calculations.

The bulk electronic structure of Co_3_Sn_2_S_2_ through two neighboring Weyl points and the bulk Fermi surfaces are shown in Figure 1C–E and agree with previously reported works.^30,48,49^ To examine the role of the surface terminations, we need to characterize this material in both real and reciprocal space. Previous STM measurements report three possible cleavage planes along the (001) direction, giving rise to three distinct situations: Co–Sn-, Sn-, and S-terminated surfaces^43^ (see Figure 1B). The former is reported to have a small lateral size of the order of a few nanometers, significantly rarer than the other planes, with the highest concentration of impurities. We will therefore focus on the S and Sn terminations, which could be accessed by photoelectron spectroscopy and provide a suitable platform for studying the effect of the surface potential on the bulk-surface connectivity of the WPs. To investigate these terminations, we used micro- and angle-resolved photoelectron spectroscopy (micro-ARPES). The small light spot of micro-ARPES, i.e., 4 μm^2^ from a capillary mirror, allowed us to probe the single/pure termination sample area, thus ensuring the absence of a mixed spectroscopic signal. In panels A and B of Figure 2, we show the spatially resolved map (each pixel is 4 μm^2^) where a part of the cleaved sample is measured. The color-code intensity maps denote where the S (Figure 2A) and Sn (Figure 2B) signals are prominent. [In the scale, red means more signal and blue means less signal. The plots have been obtained by rastering the same map and selecting the energy corresponding to the core levels of (A) S and (B) Sn.] Our maps are consistent with the STM studies in refs (43) and (44) and corroborate the previous observation that the Co-terminated areas are much smaller than our spatial resolution. From the maps in panels A and B of Figure 2, we selected two points where the S and Sn intensities were the highest, and we measured accordingly the core levels present in the sample. Consistent with our real space plots, region 2 in Figure 2B shows the largest contribution of the Sn 4d core levels (Figure 2C), as well as a significantly different shape and a smaller contribution for the S 2p core levels, compared to region 1 of Figure 2A. The latter shows also a clear surface component shifted toward a lower binding energy (Figure 2E).

**Figure 2:**
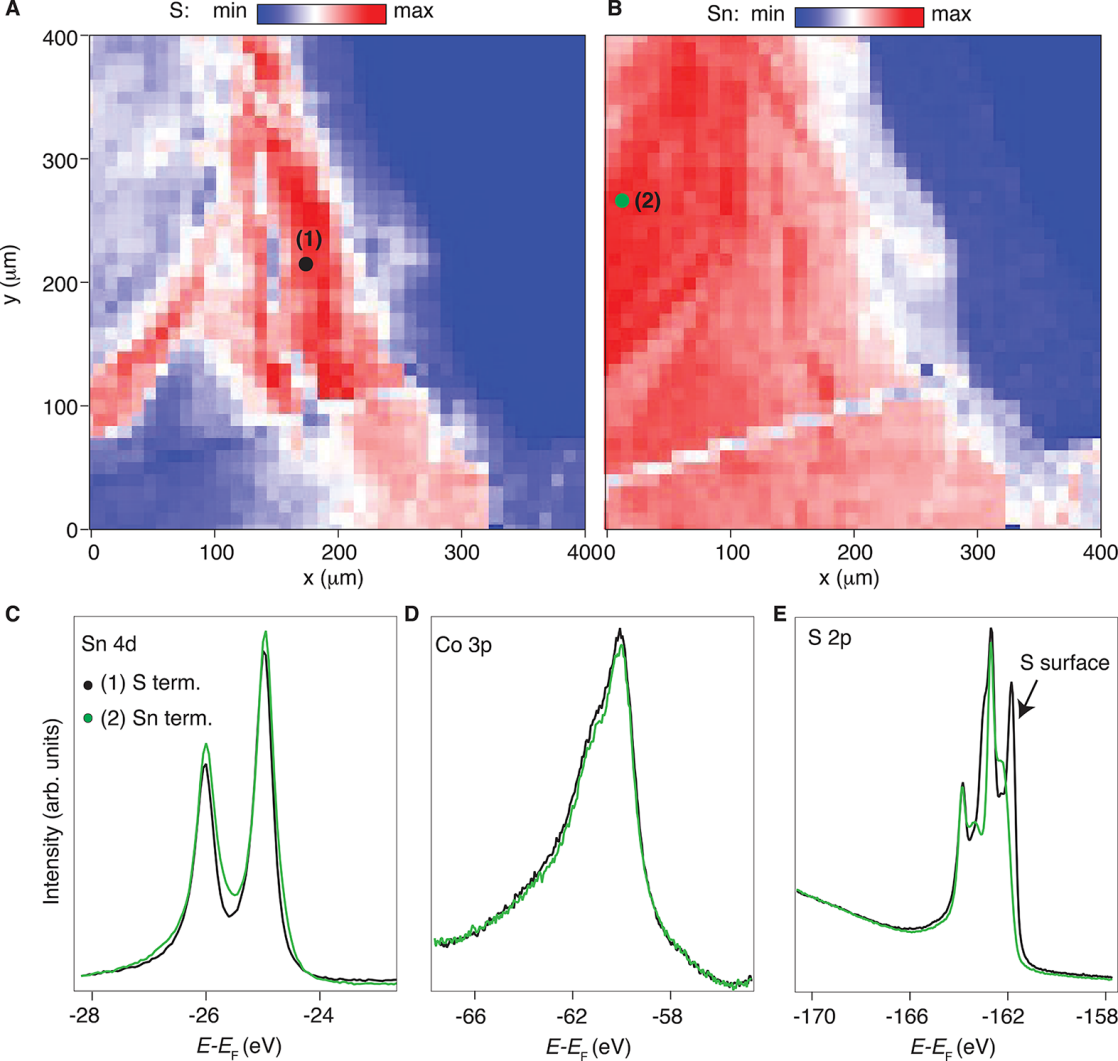
(A) Spatial map of a portion of the cleaved sample with a red color corresponding to the S termination. (B) Same as panel A but for Sn termination. (C) Sn 4d, (D) Co 3p, and (E) S 2p core levels taken from points 1 and 2, as indicated in maps A and B. For the core levels, to better allow us to make a direct comparison, the spectra have been normalized to their tails, i.e., such that for regions 1 and 2 each core level has a matching tail. In the color maps, the darkest blue regions are areas outside of the sample’s surface.

This is in contrast to what has been previously reported in ref (47), where there is no evidence of surface core level shifts. We stress that a sharp surface-derived signal is crucial for addressing the surface state manifold, the intensity of which could be otherwise smeared out by aging effects or by the presence of spectroscopic features coming from small neighboring patches of the sample. The Co 3p core levels (Figure 2D) showed a much less pronounced difference between the regions. In summary, our micro-ARPES data prove the existence of two main surfaces in Co_3_Sn_2_S_2_ with an average 40–50 μm^2^ lateral size for the S and Sn surfaces (Figure 2).

To unveil the differences between the two terminations from regions 1 and 2 in Figure 2, we acquired the energy-momentum spectra by using micro-ARPES along both the Γ–K and Γ–M directions. In Figure 3, we compare these to the DFT-calculated spectra (see the method sections for experimental conditions and details on the DFT calculations). The availability of micro-ARPES allows us also to make a direct comparison to the data collected by using “standard” ARPES (see Figure S1a,c).

**Figure 3:**
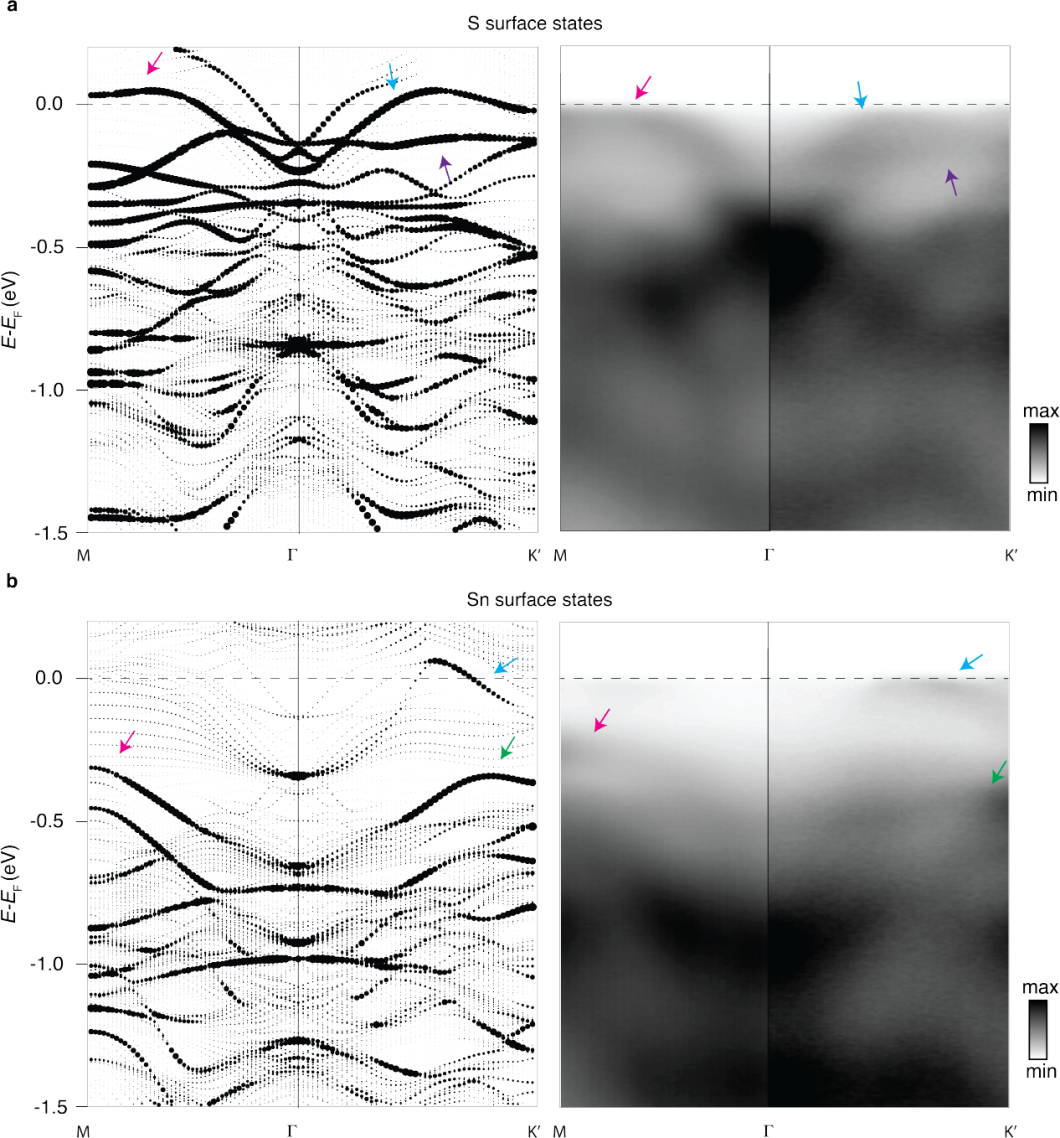
(a) DFT-calculated energy-momentum spectrum for the S termination (left) and the corresponding ARPES measurement (right) along the high-symmetry directions M, Γ, and K′. (b) DFT-calculated energy-momentum spectrum for the Sn termination (left) and the corresponding ARPES measurement (right) along the high-symmetry directions M, Γ, and K′. The point size is given by the surface character of the respective termination. The arrows serve to highlight the most prominent common features between the calculations and the ARPES. As one can see, DFT reproduces extremely well the spectroscopic features seen in ARPES at the Fermi level.

We emphasize that the theoretical results are obtained via DFT for a 65 Å supercell in the (001) direction, which corresponds to five conventional unit cells. An *ab initio* treatment of the finite size geometry incorporates structural changes and the surface potential correctly, as opposed to non-self-consistent slab models based on bulk tight-binding parameters as in refs (25), (27), (30), (43), (46), and (48). For the Sn-terminated surface, these effects appear to be of minor importance as both methods yield qualitatively similar band structures and Fermi surfaces (see Figure S2). Instead, we find significant differences in the case of S termination, where the DFT results in better agreement with the experimental data around the Fermi level (see Figure S3).

The DFT band structure in Figure 3 shows the surface states, where the dot sizes indicate the surface localization. According to the DFT, the S termination presents a surface state manifold characterized by a large electron pocket at the center of the Brillouin zone and by a smaller pocket with a minimum at approximately 100 meV. (This feature is more visible in the Supplementary Information, for more favorable matrix elements in the data collected by “standard” ARPES. Here it is present but with a significantly weaker intensity.) The larger pocket flattens significantly along the Γ–K and Γ–M directions, approaching the Fermi level. This feature interferes with the other surface states, which were already revealed by the Wannier approach. Because they possess a different degree of surface localization in the DFT calculation, a convenient way of highlighting the interesting bands giving rise to the Fermi arcs is a projection onto atoms of the second kagome layer (see Figure S4). The Sn termination, in comparison, hosts surface states that cross *E*_F_ away from the zone center. These are readily revealed by both DFT and micro-ARPES in Figure 3. Contrary to the bands reported in ref (47), our band structures for the two terminations can clearly be distinguished by various features. This strengthens the identification based on our core level analysis, for which a good agreement between the experimental and DFT band structure is observed.

We note that the data collected by “standard” ARPES on of the Sn cleavage (see Figure S1) show an intense state with electron-like dispersion at the center of the Brillouin zone. Here, we also notice how such a state matches well with the calculated electronic structure apart from an approximately 100 meV rigid shift of the calculated spectra toward the Fermi level position.

The combination of experiment and theory allows us to reveal a surface-dependent electronic structure with surface states exhibiting a markedly different dispersion, including both trivial and in-gap topological states. The curvature/flatness of these states is influenced by the type of termination; thus, the connection of the Weyl points and the local distribution of the Berry phase might also be different for the two cases.

The Fermi surfaces of Co_3_Sn_2_S_2_ for the S and Sn terminations have been collected with micro-ARPES under the same experimental conditions, and they have been compared to the *ab initio* Fermi surfaces calculated by using DFT. In Figure 4, we show both experimental and calculated results. We stress that the three-dimensional nature of Co_3_Sn_2_S_2_ is responsible for broadening the electronic structure and causes strong variations in the photoemission intensity with varying photon energies. Thus, our combined approach, involving both theory and experiment, is crucial for identifying the main components of the electronic structure.

**Figure 4:**
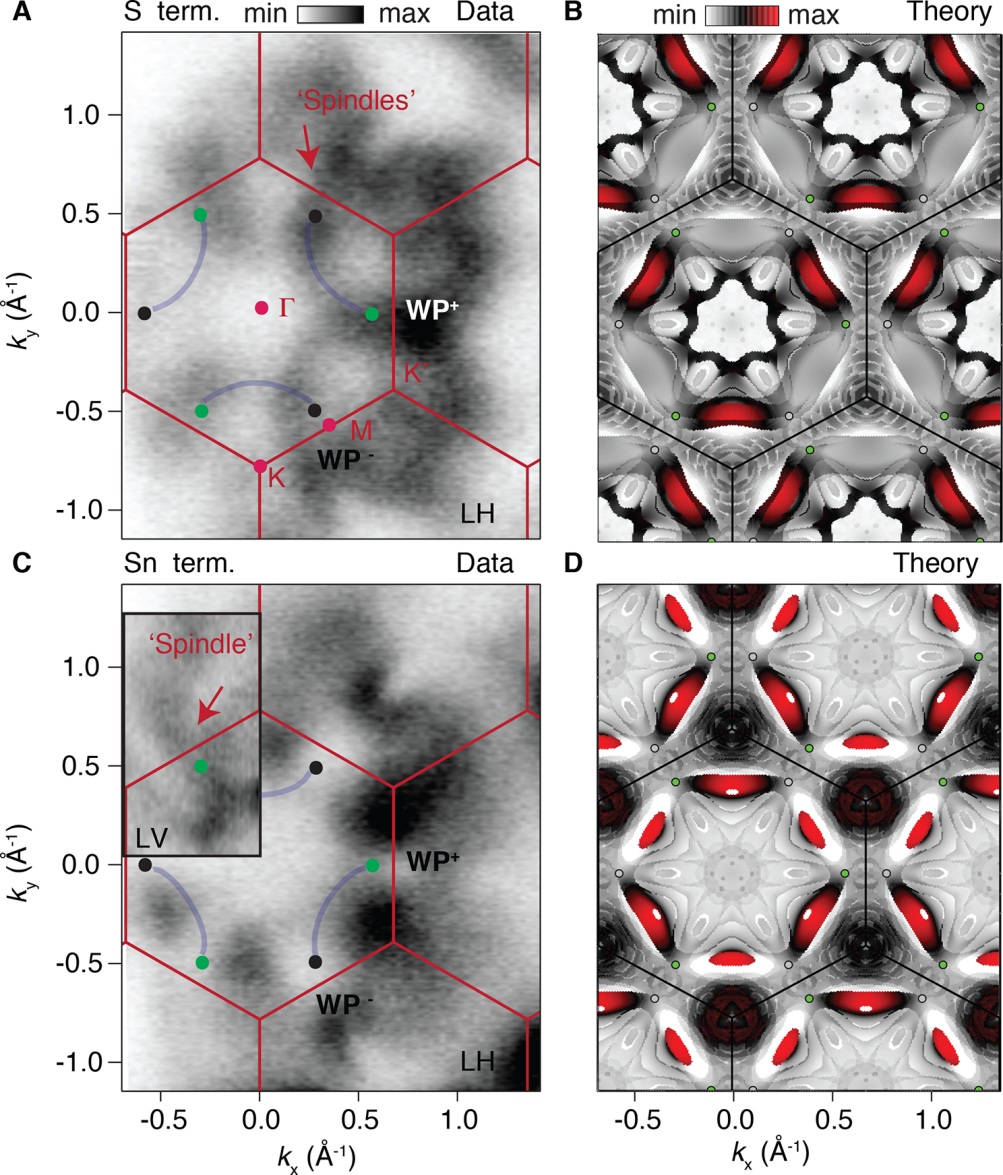
(A) Fermi surface map collected at 20 K by using micro-ARPES from region 1 belonging to the S termination with linear horizontal polarization and (B) corresponding surface map around 70 meV in a 30 meV range calculated by DFT with projections onto the second kagome layer. (C) Fermi surface map obtained by micro-ARPES for the Sn termination with linear horizontal polarization, with the inset showing a detail of the bulk-derived “spindles”, obtained by linear vertical polarization. (D) Corresponding DFT S termination at 70 meV in a 30 meV range with projections on the first kagome layer. The photon energy used was the same, i.e., 120 eV. Fermi surfaces with linear vertical polarization are available in the Supporting Information. The Weyl points are also reported together with the expected arcs.

Co_3_Sn_2_S_2_ exhibits elongated features along the Γ–M direction, bridging neighboring Brillouin zones. We will refer to them as “spindles”.^25,29^ These characteristics are bulk properties and can already be observed for the bulk Fermi surface, as shown in Figure 1D. The spindles are also noticeable in the slab calculations (Figure 4B,D). Experimentally, we detect the spindles at both terminations yet for different intensities depending on the light polarization. For linear horizontal polarization, these spectroscopic features are the most intense at the S-terminated surface (Figure 4A). On the contrary, we do not see spindles for the Sn termination with the same light (Figure 4C). However, they are recovered by using linear vertical light, as one can see in the inset of Figure 4C, where a portion of the Fermi surface under such a favorable condition has been illustrated (see also Figure S5). Already the difference with regard to the bulk-like features further proves the presence of the two distinct terminations Sn and S, as identified by the core level analysis.

Next we focus on characteristics originating from the surface states. The DFT surface maps shown in panels B and D of Figure 4 are obtained at an energy close to the Weyl points to identify their connectivity. The S-terminated surface map is dominated by the presence of trivial surface states of the large pocket. Projecting, however, on the second Co kagome layer, we strongly suppress such trivial states, highlighting the actual Weyl connectivity around the K point in Figure 4B. The projection on the first surface layer can be seen in Figure S12B. The linear vertical polarization is a good condition under which to observe surface states in this case (see Figure S5A,LV). The combination of trivial and nontrivial surface states and spindles results in an overall hexagonal pattern in the middle of the BZ (Figure S5A,LV). Experimentally, it is difficult to detect all of the theoretically predicted surface states. This is challenged not only by the strongly varying matrix elements (see Supporting Information) and the complexity of the electronic structure of this system itself but also by the presence of bulk bands (due to their large *k*_*z*_ dispersion). These appear to be very broad and often make the identification of the surface states (which are generally sharper in nature) difficult. The identification of the main features in panels A and B of Figure 4 is possible thanks to comparison with DFT. However, the complexity of the system and the strongly varying matrix elements make it very challenging to understand by ARPES the subtle differences observed from K and K′. In the Supporting Information (Figure S8), we have also shown additional data collected closer to the photon energy corresponding to the Weyl point, and we could, in such a configuration, identify a 3-fold symmetric pattern, like that predicted by DFT. The latter allows us to identify the spindles and the intra-BZ connections of the Weyl points with Fermi arcs around the K points (and not K′).

In contrast, the Sn termination shows a Fermi surface with spectroscopic features mainly located around the K and K′ points of the BZ for linear horizontal polarization. Such characteristics are expected on the basis of the triangular surface state from DFT and contribute to the spectral weight with the largest signal (Figure 4C). Additionally for the Sn termination, our DFT shows arcs that connect the WPs in an “opposite fashion” compared to the S termination, winding around the K′ points (and not K). The pairing can also be obtained from the surface band structure through adjacent Weyl points, as shown in Figure S11, where we characterize nontrivial surface states linking the Weyl points. For better identification of the Weyl point connection, we utilized a rather large energy interval of 30 meV for the surface maps. In Figure S12, we depict typical Fermi arcs on surface maps by using a smaller energy range. Also in the Sn-terminated case, experimentally we see an overall hexagonal pattern with intensity connecting the WPs, due to the combination of trivial surface states and Fermi arcs (see Figure S5B,LV).

The Fermi surfaces were collected under the same conditions to better appreciate the spectroscopic differences observed between the S and Sn terminations. The experimental Fermi surfaces show both trivial and nontrivial surface states that connect the Weyl points. Even though by changing the photon energy, it is possible to enhance the intensity of the surface states, resulting in their simpler identification (see Figure S5), and despite the one-to-one comparison between calculations and ARPES being good overall, it is still challenging to distinguish between trivial and nontrivial surface states. However, the evident differences observed in our data are still important to show how the surface electronic environment is crucial for modulating both topological and topologically trivial components (Supporting Information).

In summary, the two surface cleavage planes give a markedly different Fermi surface for Co_3_Sn_2_S_2_. The importance of these findings has a direct link to the development of highly controllable devices, where the electrostatic potential can be used to finely modulate the local topology. We additionally mention that experimentally and theoretically the Sn termination is in agreement with previous works^30,46^ (see Figure S7). In this study, micro-ARPES allowed us to differentiate unambiguously the S and Sn surfaces to prove that these terminations host significantly different Fermi surfaces with distinctive connectivity. Depending on the termination, the Fermi arcs connect different Weyl points inside the BZ.

Our results prove that the surface environment is crucial for determining the shape of the arcs connecting the WPs, thus revealing that the bulk-surface connectivity is strongly altered by the type of termination. This situation is similar to what happens in polar materials;^50,51^ however, in the case presented here, it has implications in the k-resolved local topology, which is determined by the fashion in which the WPs are connected, as shown in ref (43).

In conclusion, by using micro-ARPES and DFT calculations, we not only verified the existence of two micrometer size terminations on Co_3_Sn_2_S_2_ but also unambiguously demonstrated how in this compound the same bulk topology gives rise to striking differences in the Weyl point connectivity on each of the two surface terminations. To prove this, it was crucial to collect both core levels, Fermi surfaces, and energy-momentum spectra from the same micrometric and single-domain surface area. In Co_3_Sn_2_S_2_, we show visibly different topological surface states dispersed across the Fermi energy. Here, we shed light on the possibility of modulating the surface electronic environment of Co_3_Sn_2_S_2_ by using electrostatic potentials to shape the transport properties of this system, *in primis* dictated by the interplay of SOC and magnetism. Potential future implications of this study are expected to emerge in the field of designer heterostructure design.

## Supporting Information Available

Additional ARPES data and DTF calculations that are useful for understanding the results of the main text, in particular, *k*_*z*_ scans as well as data collected with various polarizations of the incoming photons (PDF)
