# Supplementary material for: Observation of Termination-Dependent Topological Connectivity in a Magnetic Weyl Kagome Lattice
Source: Nano Lett. 2023 Aug 28;23(17):8035–42. doi: 10.1021/acs.nanolett.3c02022 (PMC10510577; doi:10.1021/acs.nanolett.3c02022)
Supplement: Supplementary file 1 — nl3c02022_si_001.pdf [file nl3c02022_si_001.pdf]

Supplementary Information: Observation of termination-dependent topological connectivity in a magnetic Weyl kagome-lattice

Federico Mazzola,[*,†] Stefan Enzner,[¶] Philipp Eck,[¶] Chiara Bigi,[§] Matteo Jugovac,[‖] Iulia Cojocariu,[‖] Vitaliy Feyer,[‖] Zhixue Shu,[⊥] Gian Marco Pierantozzi,[‡] Alessandro De Vita,[#] Pietro Carrara,[#] Jun Fujii,[‡] Phil D. C. King,[§] Giovanni Vinai,[‡] Pasquale Orgiani,[‡] Cephise Cacho,[@] Matthew D. Watson,[@] Giorgio Rossi,[#] Ivana Vobornik,[‡] Tai Kong,[⊥] Domenico Di Sante,[*,△] Giorgio Sangiovanni,[*,¶] and Giancarlo Panaccione[*,‡]

†*Department of Molecular Sciences and Nanosystems, Ca' Foscari University of Venice, 30172 Venice, Italy*

‡*Istituto Officina dei Materiali, Consiglio Nazionale delle Ricerche, Trieste I-34149, Italy*

¶*Institut für Theoretische Physik und Astrophysik and Würzburg-Dresden Cluster of Excellence ct.qmat, Universität Würzburg, 97074 Würzburg, Germany*

§*School of Physics and Astronomy, University of St Andrews, St Andrews KY16 9SS, United Kingdom*

‖*Elettra Sincrotrone Trieste S.C.p.A., S. S. 14, km 163.5, 34149 Trieste, Italy*

⊥*Department of Physics, University of Arizona, Tucson, AZ 85721, USA*

#*Dipartimento di Fisica Universitá di Milano, Via Celoria 16, Milano 20133, Italy*

@*Diamond Light Source, Harwell Campus, Didcot, OX11 0DE, United Kingdom*

△*Department of Physics and Astronomy, University of Bologna, 40127 Bologna, Italy*

∇*Center for Computational Quantum Physics, Flatiron Institute, 162 5th Avenue, New York, NY 10010, USA*

E-mail: federico.mazzola@unive.it; domenico.disante@unibo.it; sangiovanni@physik.uni-wuerzburg.de; panaccione@iom.cnr.it

[+]F.M. and S.E. contributed equally to this paper.

Micro-ARPES spatial maps plotting

In order to plot the real space maps for the S and Sn terminations shown in Fig.2a-b of the main text, we did the following: We colored the maps by selecting the energies of the S (and Sn) core levels for the full area. In particular, (1) For the S termination, we identified features in the micro-ARPES spectra which were present only when the surface component of the S 2*p* core levels was visible. Then, we tracked the regions across the probed area, where such features showed up. (2) for the Sn termination, we used the same idea and we identified spectroscopic feature which were only visible when the signal of the Sn 4*d* core levels were the highest and the surface component of the S 2*p* core levels was absent. It is also worth mentioning that we have collected the same maps for Co core levels and we did observe a constant intensity except a few small areas which, however, did not gove any coherence in the ARPES spectra, likely indicating the disordered character of this surface compared to S and Sn.

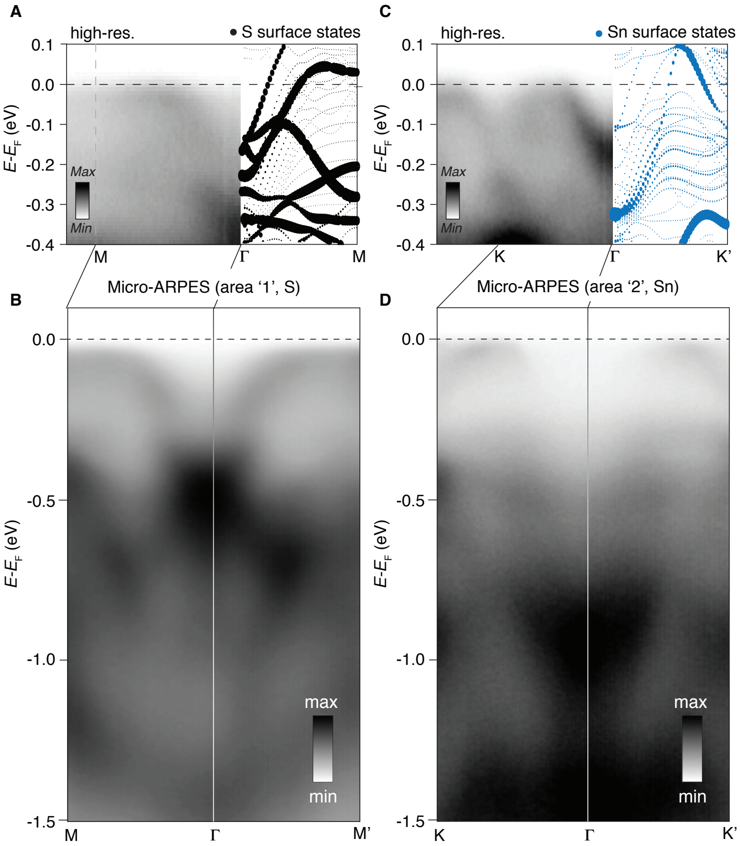

Figure 1: (A) High-resolution ARPES along the Γ-M direction and corresponding DFT calculated spectrum obtained for the S surface states. (B) Extended range obtained from spot '1' by using micro-ARPES.(C) High-resolution ARPES along the Γ-K′ direction and corresponding DFT calculated spectrum obtained for the Sn surface states. (D) Extended range obtained from spot '2' by using micro-ARPES. The measurements have been performed at at 20 K. We notice that the high-resolution data also show an electron-like pocket at the centre of the zone which is reconcilable with the DFT calculations with a simple 100 meV rigid shift towards E_F.

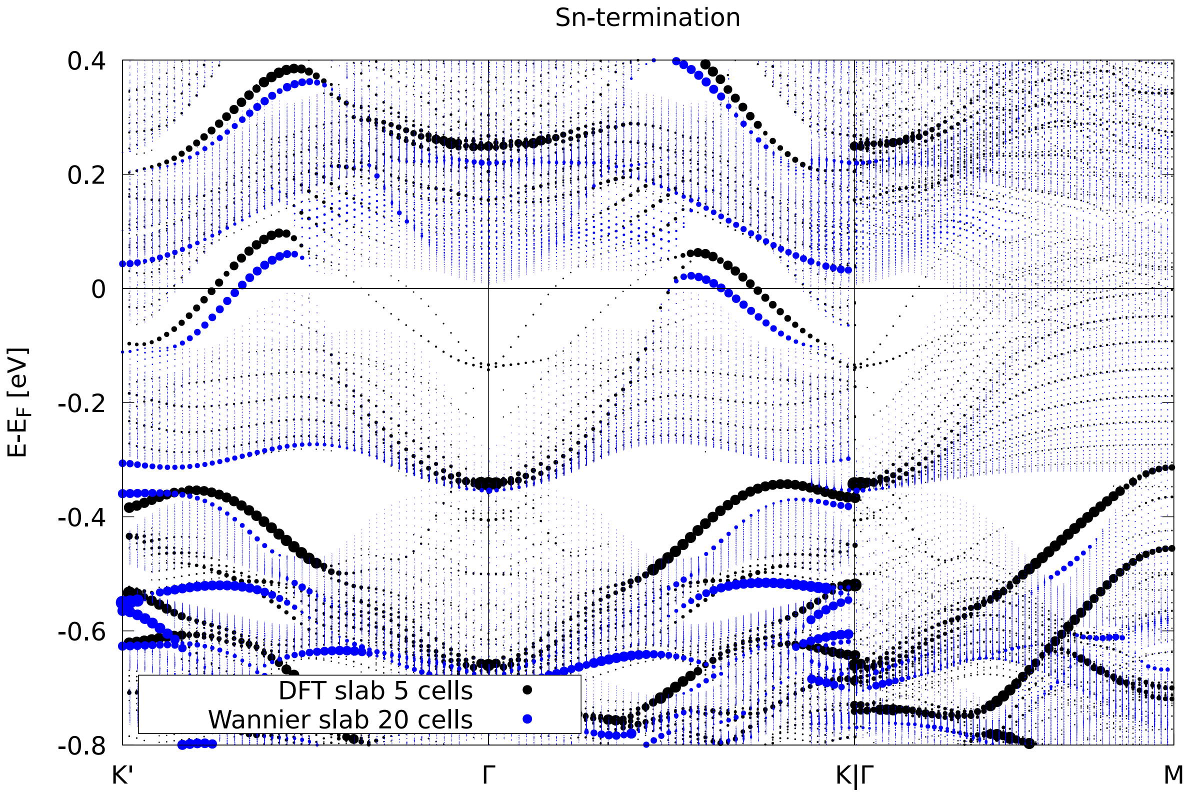

Figure 2: Comparison of surface states obtained with different methods for the Sn-termination along the K′-Γ-K|Γ-M path. The surface band structure for a supercell, consisting of five conventional cells stacked in z-direction, as calculated with DFT is shown in black. The corresponding band structure from a Wannier Hamiltonian based on the conventional unit cell is depicted in blue. The size of the dots indicates the localization in the first three layers. Differences in the Fermi energies between the two methods may arise, as the Fermi energy is not calculated self-consistently for the Wannier slab approach.

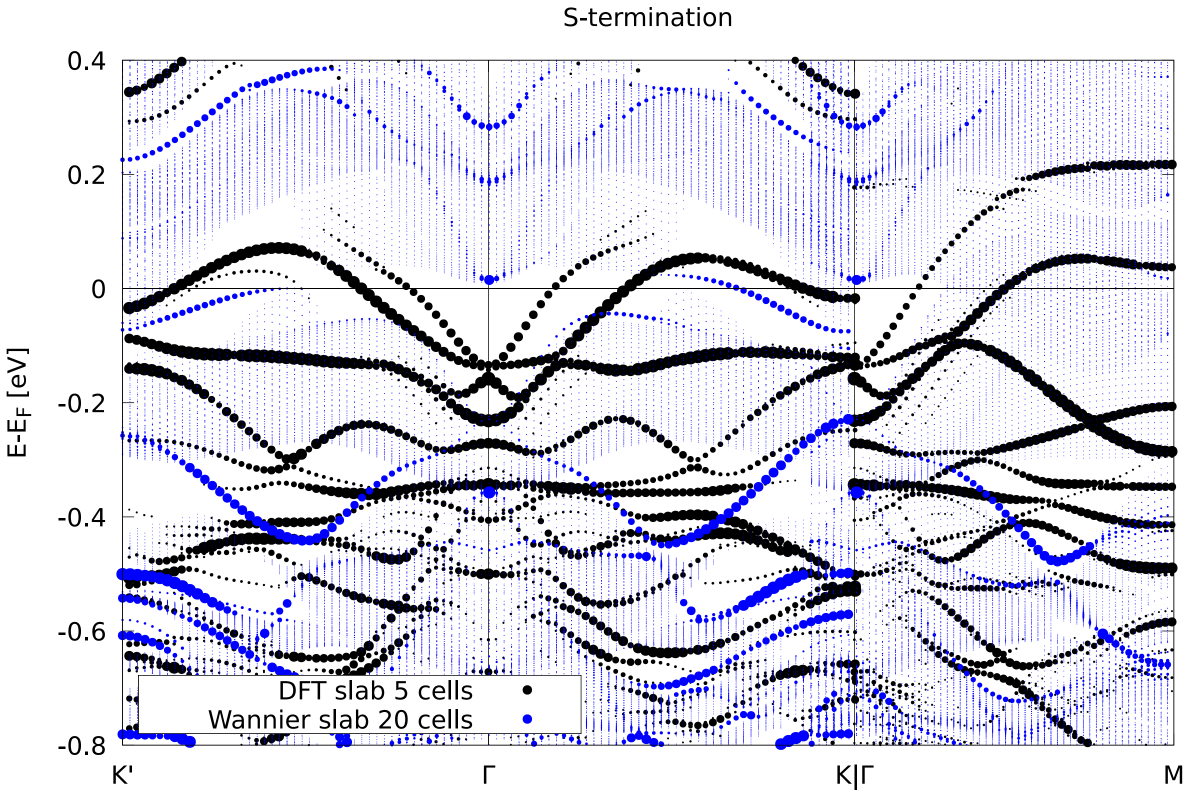

Figure 3: Comparison of surface states obtained with different methods for the S-termination along the K′-Γ-K|Γ-M path. The surface band structure for a supercell, consisting of five conventional cells stacked in z-direction, as calculated with DFT is shown in black. The corresponding band structure from a Wannier Hamiltonian based on the conventional unit cell is depicted in blue. The size of the dots indicates the localization in the first two layers. In the DFT approach we observe additional surface states not captured by the Wannier approach i.e. the pocket around the Gamma point, which becomes flat towards the K and M point.

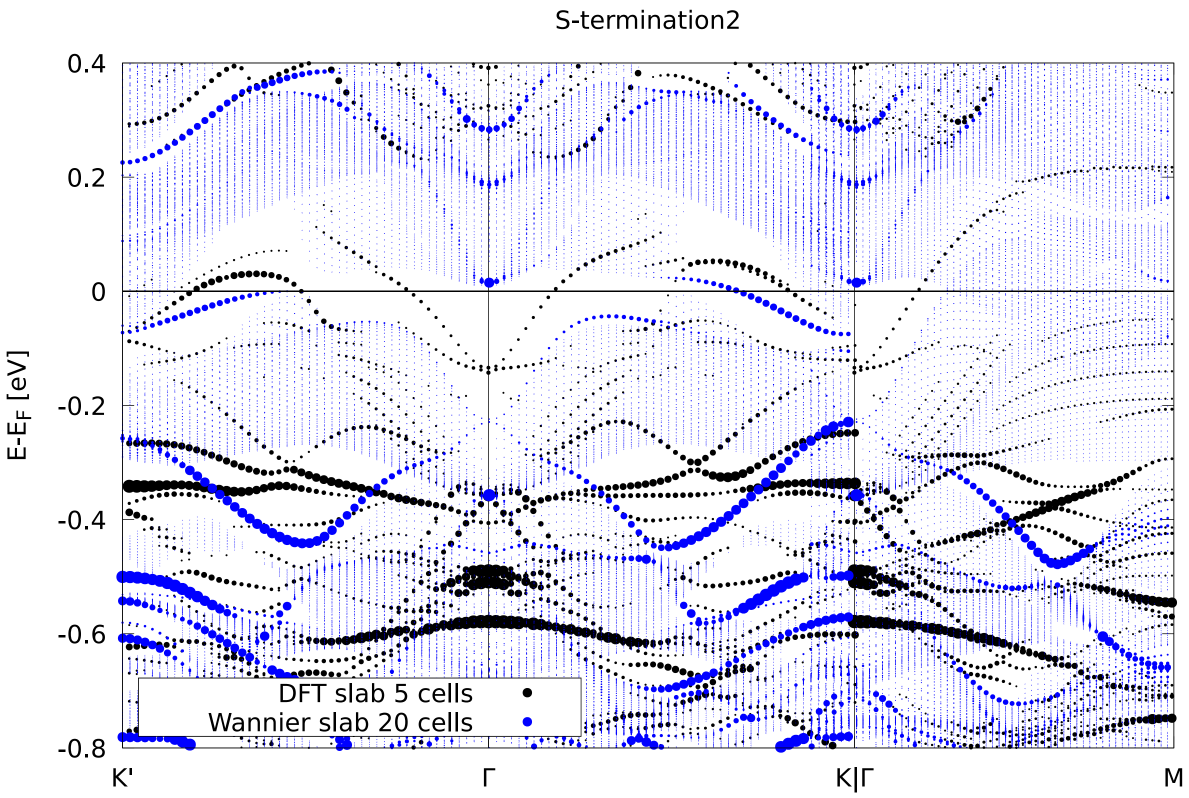

Figure 4: Comparison of surface states obtained with different methods for projections of the second outermost Co-kagome layer of the S-termination along the K′-Γ-K|Γ-M path. The surface band structure for a supercell, consisting of five conventional cells stacked in z-direction, as calculated with DFT is shown in black. The corresponding band structure from a Wannier Hamiltonian based on the conventional unit cell is depicted in blue. The size of the dots indicates the localization in the second kagome layer including adjacent S-layers. Here we exploit that the surface states, already noticed from the Wannier calculation, also localize on second Co-kagome layer, whereas the additional surface states revealed by DFT are more restricted to the first kagome layer.

Matrix elements, photon energy, and three-fold spectral weight

In this section we show additional experimental data which allow us to better understand the role of matrix elements in the electronic structure, dimensionality of the observed bands, and also help the visualization of certain features. Indeed, matrix elements are of paramount importance in photoemission experiments and can determine the intensity of the ARPES maps significantly. Such matrix elements depend on the photon energy, photon polarization, and also on the geometry of the system. In order to shed light on this aspect, here, we report the study of the electronic properties of $Co_3Sn_2S_2$ with different experimental configurations. In Fig.5, the Fermi surface maps of the system are reported from both (a) S and (b) Sn terminations from areas of $4\mu m^2$ and for both linear vertical and linear horizontal configurations. Such configurations correspond to having the light polarization vector fully in-plane and (parallel to the analyzer slit) and also 50% in-plane (orthogonal to the analyzer slit) and 50% out of plane. We notice that the data highlights a strong linear dichroism, i.e. different polarizations yield very different spectroscopic signals. Importantly, by using both polarizations we can detect the "spindles", which appear for both terminations (as in our calculations) along the Γ-M direction of the Brillouin zone. This observation is particularly important, because it allows us to exclude the presence of rotational domains, leading to the wrong attribution of the spectral weight measured. In other words, if the observed features were given by domains, the "spindles", in one termination case, would not be along Γ-M. To better visualize this, we also show for both surfaces, both light polarizations, and both Γ-K and Γ-M directions the electronic structure as energy versus momentum and we found strikingly different dispersions between S and Sn terminated samples. Finally, we also have reported the Fermi surface maps by rotating the sample by 90 degrees, and we have made sure that the features observed in Fig.5 and 6 are consistent with our attribution. We reported this in Fig.7.

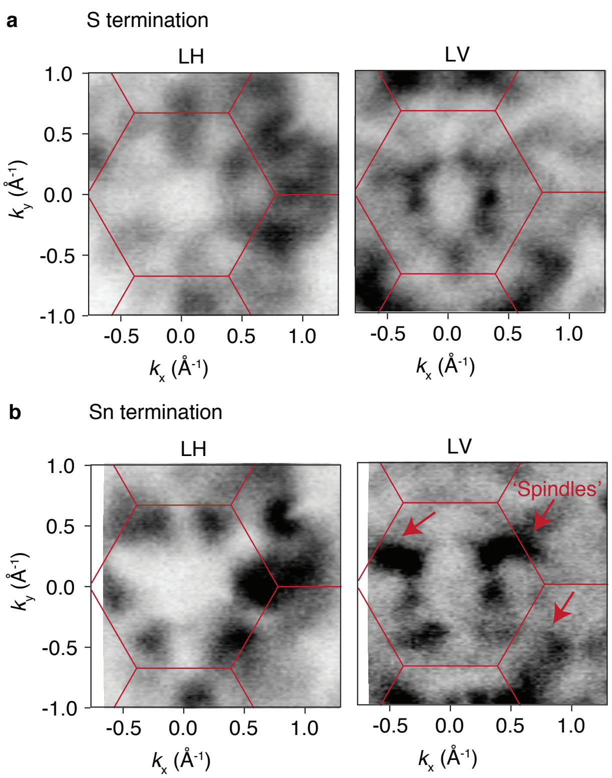

Figure 5: (a) S termination Fermi surface collected with linear horizontal (left) and vertical (right) light. (b) Sn termination Fermi surface collected with linear horizontal (left) and vertical (right) light.

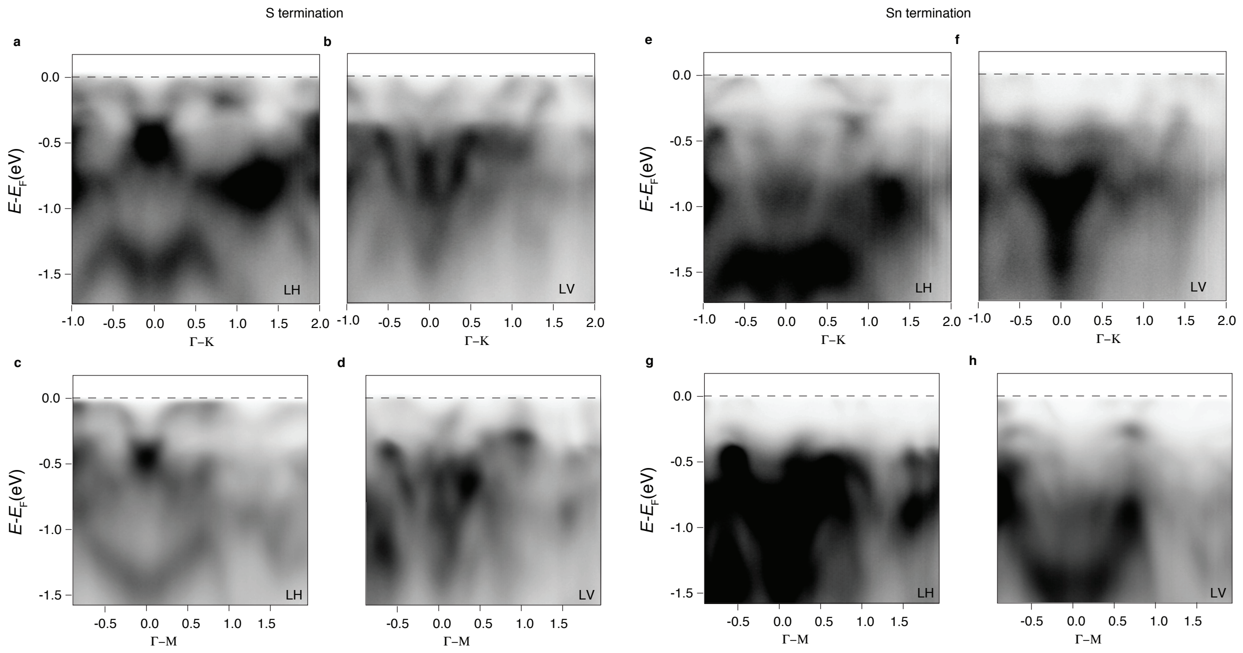

Figure 6: S termination collected at 120 eV with (a) linear horizontal and (b) linear vertical polarization along the and along the Γ-K direction. The same collected with (c) linear horizontal and (d) linear vertical polarization along the and along the Γ-M direction. Sn termination collected at 120 eV with (e) linear horizontal and (f) linear vertical polarization along the and along the Γ-K direction. The same collected with (g) linear horizontal and (h) linear vertical polarization along the and along the Γ-M direction.

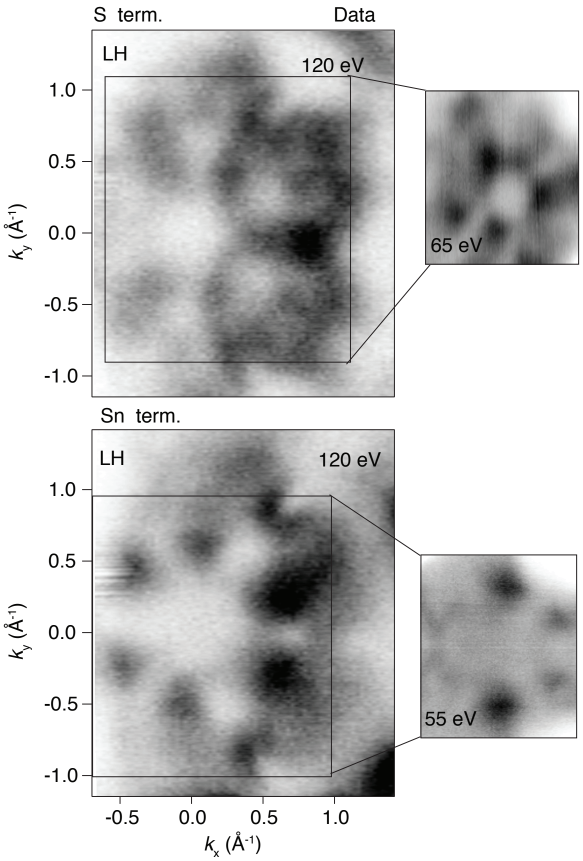

Figure 7: Termination-dependent Fermi surface maps collected with linear horizontal light after the sample has been rotated by 90 degrees compared to Fig.5. We notice that for both terminations, the spectral features remain the same.

In order to give a comprehensive understanding of the electronic properties of this system, we also show in the following photon energy dependent scans collected for one termination (Sn-termination) showing the two-dimensional character of the states observed. Also, as an example, we selected an energy close to the Weyl points (137 eV) at which the three-fold symmetric pattern expected for a system where K and K' are not equivalent, shows up better and where the features that we compare to the DFT in the main text are better visible (See Fig.8).

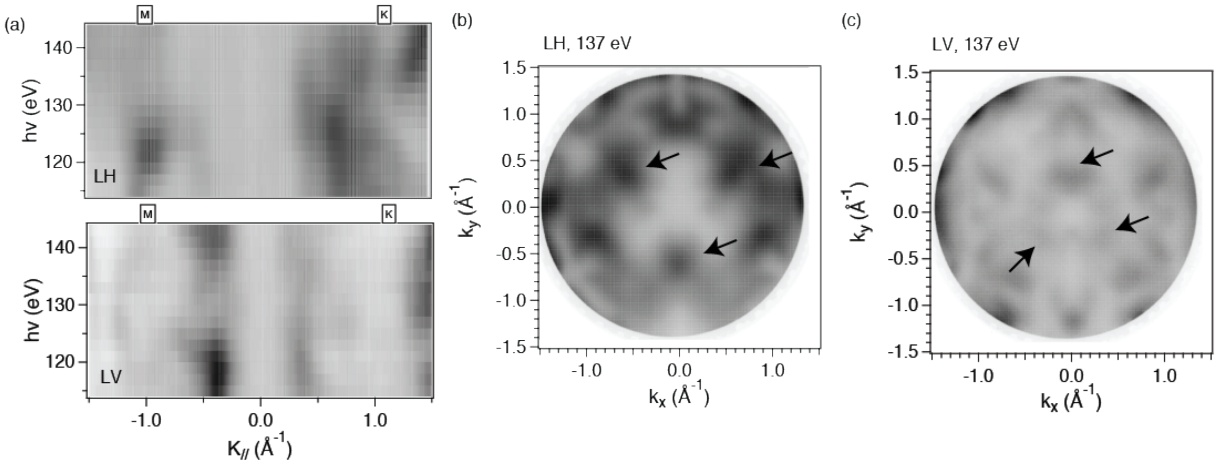

Figure 8: (a) Photon energy scan collected for the Sn-termination with both LH and LV polarization. The stripe-like behaviour is in agreement with previous works and indicates the two-dimensional character of the electronic states. Selected photon energy (137 eV) close to the Weyl points for (b) LH and (c) LV polarization where the three-fold pattern (due to K and K' difference) is more prominent. The three-fold symmetric intensity is also indicated by the arrows.

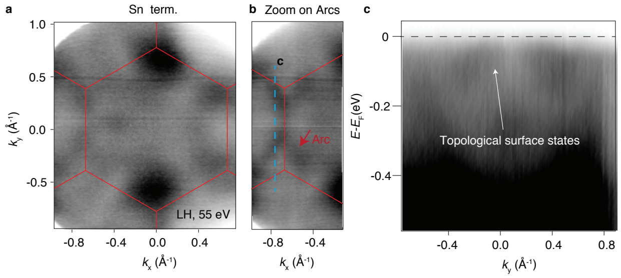

Figure 9: (a) Sn termination Fermi surface collected with linear horizontal polarization and at 55 eV and its corresponding (b) zoom on the Fermi arcs. Such arcs form a triangular patter around the K point of the Brillouin zone. (c) Energy-momentum spectra extracted along the blue dashed line in (b) and showing the topological surface states.

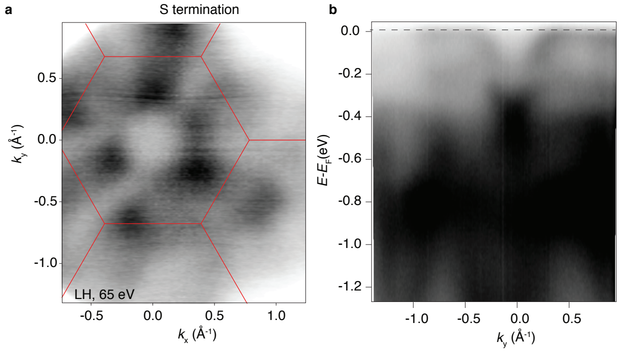

Figure 10: (a) S termination Fermi surface collected with linear horizontal polarization and at 65 eV and its corresponding (b) energy-momentum dispersion along Γ-M. The high resolution data are in perfect agreement with those obtained by nano-ARPES and provide a complementary view of the states belonging to the Sn surface manifold.

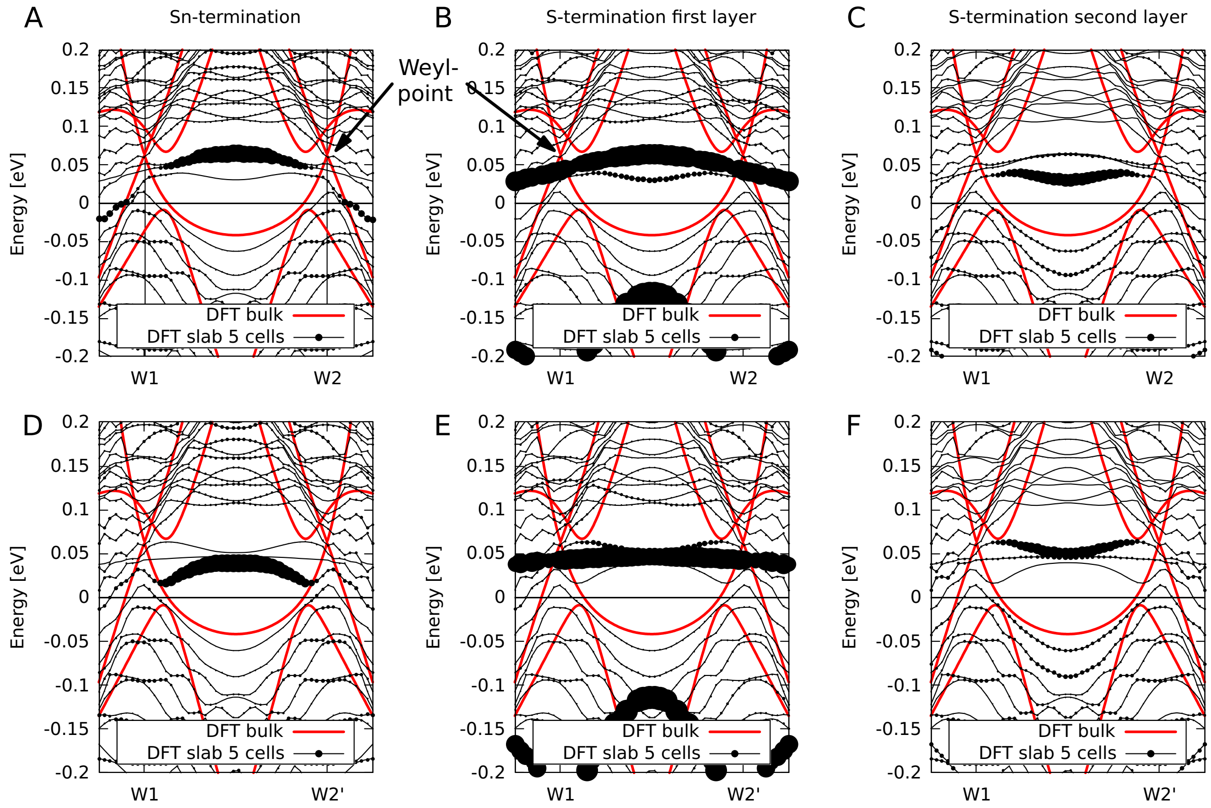

Figure 11: (A-F) DFT slab band structure along different Weyl-point overlaid with the corresponding bulk band structure. The point size corresponds to the projection on the Sn-terminated surface and on the S-terminated surface for the first and second Co-kagome layer individually. For (A) and (F) non-trivial surface states can be identified, approximately connecting the Weyl-points at 60 meV indicated by W1, W2 and W2'. W2 and W2' represent the two neighbouring Weyl-point in the Brillouin zone of Weyl-point W1. See Fig. 12D.

Supplementary Information Methods

The samples were grown by a self-flux method.[1] Starting elements were loaded in an alumina crucible in a stoichiometric ratio and sealed in a silica tube under vacuum. The silica ampule was then heated to 1000 °C, kept there for 24 hours and slowly cooled to room temperature. Micro-ARPES measurements were performed at the I05 beamline at Diamond Light Source, UK. A capillary mirror optic was used to focus the beam to $\sim$4 μm, and a DA30 hemispherical analyser was used to measure the photoelectrons with a combined energy resolution of 30 meV. Fermi surface maps were collected by rotating the analyser around the fixed sample position. The 'standard' ARPES has been performed at the APE-LE laboratory of NFFA facility of IOM-CNR at the Elettra synchrotron radiation source (Trieste, IT - https://www.nffa.eu) by using a DA30 hemispherical analyser and also at the NanoEsca laboratory of the Elettra synchrotron. All the photoemission data have been collected at $\approx$ 20 K, temperature well below the sample's ferromagnetic transition (T_C=175 K) and at the base pressure of 1×10^{-10} mbar

The DFT calculations of magnetic $Co_3Sn_2S_2$ were performed as implemented in the Vienna *ab initio* simulation package (VASP),[2] within the projector augmented-plane-wave (PAW) method.[3,4] For the exchange-correlation potential the PBE-GGA functional[5] was used, by expanding the Kohn-Sham wave functions into plane waves up to an energy cutoff of 270 eV. We sample the Brillouin zone on a $8 \times 8 \times 1$ Monkhorst-Pack mesh and employ spin-orbit coupling self-consistently.[6]

To investigate the electronic structure of the surface we calculate a slab geometry of five conventional unit cells in z-direction with a vacuum distance of 15 Å. All atoms in the outer layers are allowed to relax in non-periodic direction until forces were below 0.01 eV/Å. At the S-termination the magnetic moments for the Co-atoms were constrained to match the magnetic moments in the bulk region.

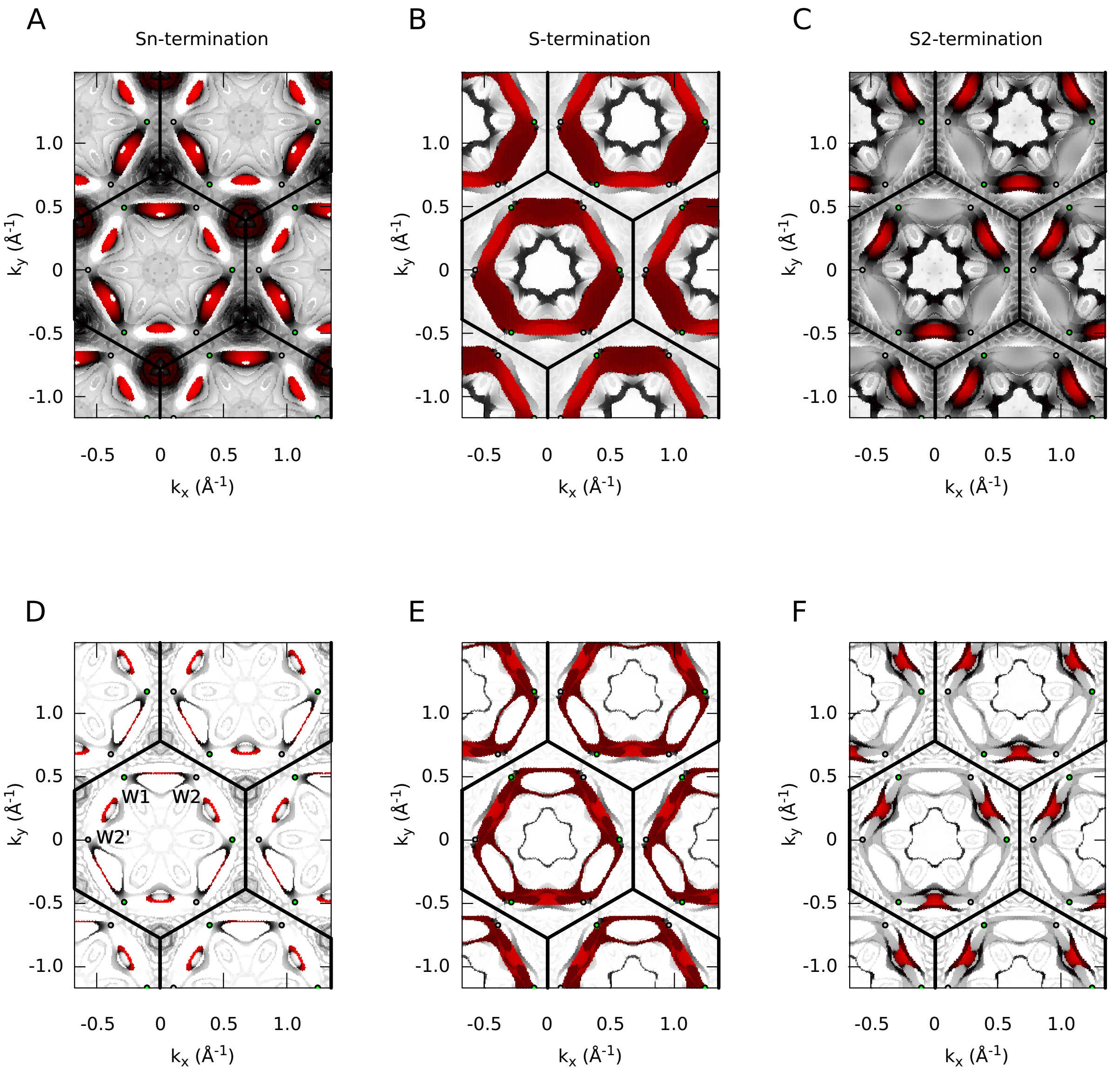

Figure 12: (A-F) DFT Fermi surface maps for different terminations and energy integration ranges. For (A-C) a energy window of 30 meV was used around 70 meV above the Fermi level to capture the surface band around the Weyl-energy. In (D-E) the energy range was reduced to 10 meV around 50 meV in order to show the typical Fermi arc representations in D and F. The states most dominant in B and E are trivial surface states. The surface character is indicated for increasing intensity from white, black to red color.

The magnetic moments of the cobalt atoms were 0.338 μ_{B} in the bulk DFT calculation, which was used for the Wannier model. The tin atoms showed small magnetic moments on the order of -0.020μ_{B} to -0.030μ_{B}. The magnetization was exclusively perpendicular to the kagome layers.

In the DFT supercell slab calculation of five conventional unit cell in the z-direction, the magnetic moments of the cobalt atoms in the bulk region were found to be 0.34μ_{B} to 0.35μ_{B}. The S-terminated surface exhibit significantly reduced magnetic moments, however they were constrained to the values of the bulk region.

For the bulk Fermi surfaces in Fig.1 of the main text we project the Bloch wave functions of the conventional bulk cell onto maximally localized Wannier functions based on Co $3d$, Sn $5p$ and S $4p$ orbitals and construct a tight-binding Hamiltonian using the WANNIER90 package.[7] We calculate bulk Fermi surfaces using the Green's function method with WannierTools.[8] The Fermi surfaces shown in Fig.3 of the main text where done with DFT in VASP. Solely for comparison to our supercell DFT approach, we obtain the band structure of a Wannier slab. (See supplementary Fig.S2, Fig.S3 and Fig.S4).

The input files of the calculations can be found in https://dx.doi.org/10.17172/NOMAD/2023.08.25-2.

References

(1) Liu, D. F. et al. Magnetic Weyl semimetal phase in a Kagome crystal. *Science* **2019**, *365*, 1282–1285.

(2) Kresse, G.; Furthmüller, J. Efficient iterative schemes for ab initio total-energy calculations using a plane-wave basis set. *Phys. Rev. B* **1996**, *54*, 11169–11186.

(3) Kresse, G.; Joubert, D. From ultrasoft pseudopotentials to the projector augmented-wave method. *Phys. Rev. B* **1999**, *59*, 1758–1775.

(4) Blöchl, P. E. Projector augmented-wave method. *Phys. Rev. B* **1994**, *50*, 17953–17979.

(5) Perdew, J. P.; Burke, K.; Ernzerhof, M. Generalized Gradient Approximation Made Simple. *Phys. Rev. Lett.* **1996**, *77*, 3865–3868.

(6) Steiner, S.; Khmelevskyi, S.; Marsmann, M.; Kresse, G. Calculation of the magnetic anisotropy with projected-augmented-wave methodology and the case study of disordered $Fe_{1-x}Co_x$ alloys. *Phys. Rev. B* **2016**, *93*, 224425.

(7) Mostofi, A. A.; Yates, J. R.; Lee, Y.-S.; Souza, I.; Vanderbilt, D.; Marzari, N. wannier90: A tool for obtaining maximally-localised Wannier functions. *Computer Physics Communications* **2008**, *178*, 685–699.

(8) Wu, Q.; Zhang, S.; Song, H.-F.; Troyer, M.; Soluyanov, A. A. WannierTools : An open-source software package for novel topological materials. *Computer Physics Communications* **2018**, *224*, 405 – 416.
